# How have smallholder farmers used digital extension tools? Developer and user voices from Sub-Saharan Africa, South Asia and Southeast Asia

**DOI:** 10.1016/j.gfs.2021.100577

**Published:** 2022-03

**Authors:** Sam Coggins, Mariette McCampbell, Akriti Sharma, Rama Sharma, Stephan M. Haefele, Emma Karki, Jack Hetherington, Jeremy Smith, Brendan Brown

**Affiliations:** a School of Engineering, Australian National University, Acton, ACT, 2600, Australia; b Australian Centre for International Agricultural Research, 38 Thynne Street, Bruce, ACT, 2617, Australia; c Knowledge, Technology and Innovation Group, Wageningen University, Droevendaalsesteeg 4, 6708, PB, Wageningen, the Netherlands; d International Maize and Wheat Improvement Center, Khumaltar, Lalitpur, 44700, Nepal; e Department of Sustainable Agriculture Sciences, Rothamsted Research, West Common, Harpenden, AL5 2JQ, United Kingdom; f The Centre for Global Food and Resources, University of Adelaide, 10 Pulteney Street, SA, 5005, Australia

**Keywords:** Participatory, Advisory, Agriculture, Affordance, Socio-technical, Gender

## Abstract

Digital extension tools (DETs) include phone calls, WhatsApp groups and specialised smartphone applications used for agricultural knowledge brokering. We researched processes through which DETs have (and have not) been used by farmers and other extension actors in low- and middle-income countries. We interviewed 40 DET developers across 21 countries and 101 DET users in Bihar, India. We found DET use is commonly constrained by fifteen pitfalls (unawareness of DET, inaccessible device, inaccessible electricity, inaccessible mobile network, insensitive to digital illiteracy, insensitive to illiteracy, unfamiliar language, slow to access, hard to interpret, unengaging, insensitive to user's knowledge, insensitive to priorities, insensitive to socio-economic constraints, irrelevant to farm, distrust). These pitfalls partially explain why women, less educated and less wealthy farmers often use DETs less, as well as why user-driven DETs (e.g. phone calls and chat apps) are often used more than externally-driven DETs (e.g. specialised smartphone apps). Our second key finding was that users often made - not just found - DETs useful for themselves and others. This suggests the word ‘appropriation’ conceptualises DET use more accurately and helpfully than the word ‘adoption’. Our final key finding was that developers and users advocated almost ubiquitously for involving desired users in DET provision. We synthesise these findings in a one-page framework to help funders and developers facilitate more useable, useful and positively impactful DETs. Overall, we conclude developers increase DET use by recognizing users as fellow developers – either through collaborative design or by designing adaptable DETs that create room for user innovation.

## Highlights

• How is ICT (video, mobile apps, SMS etc) used in agricultural knowledge brokering?
• We interviewed 40 developers and 101 users of digital extension tools.
• Fifteen pitfalls commonly constrained use of digital extension tools by farmers.
• Farmers actively appropriated, not just passively adopted, digital extension tools.
• Creating room for user innovation increases use of digital extension tools.

## Graphical abstract

**Figure:**
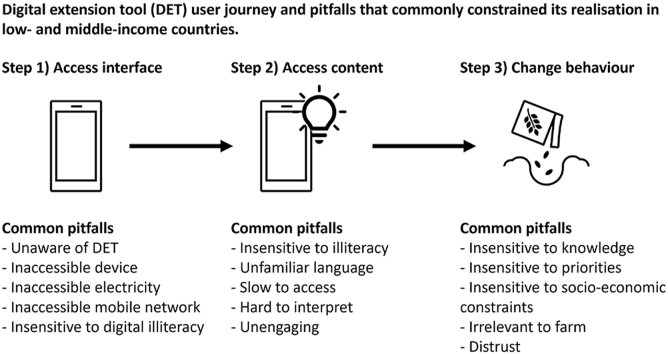

## 1 Introduction

Agricultural knowledge brokering enables global food security and other development impacts (Cui et al., 2018). However, the practice of agricultural extension is notoriously difficult to facilitate in a way that is cost-efficient (Gautam, 2000), equitable (Cunguara and Moder, 2011) and useful (Klerkx et al., 2012a). Growing accessibility of (smart)phones and mobile networks in low- and middle-income countries (LMICs) creates opportunities to address these challenges through digital extension tools (Fabregas et al., 2019).

We define a digital extension tool (DET) as a digital tool through which farmers or other extension actors share, access or discuss agricultural information or knowledge. This can include digital platforms *built* for agricultural knowledge brokering. For example, farming videos used by extension workers to discuss novel technologies with farmers (Gandhi et al., 2007), specialised smartphone apps used by farmers to diagnose crop diseases (Rupavatharam and Kennepohl, 2018) and formal voice message services used by farmers to access agronomy tips (Palmer and Darabian, 2012a). However, our DET definition also includes unstructured digital platforms *adapted* for agricultural knowledge brokering. For example, informal phone calls used by pastoralists to access information about grazing resources (Butt, 2015), YouTube channels made by farmers to offer farming advice (YouTube, 2021a, 2021b) and chat apps used by government extension workers to discuss local farming issues with peers (Munthali et al., 2018). Recognizing these less formal DETs acknowledges that agricultural extension is not necessarily an appointed role but a practice anyone may engage in (Klerkx et al., 2012b; Shove et al., 2012).

Low and socially inequitable uptake of DETs constrains their potential positive impacts. Farmers and extension actors have commonly rejected DETs, deeming them insufficiently useable (Wyche and Steinfield, 2016; Verma et al., 2014; Fawole and Olajide, 2012) or insufficiently useful (Munthali et al., 2018; Jayanthi and Asokhan, 2016). This limited uptake has prevented DETs from influencing agricultural practices (Maredia et al., 2018; Asaka & Smucker et al., 2016) and thus downstream development outcomes, like improved agricultural productivity (Fabregas et al., 2017), household incomes (Mittal and Mehar, 2012) and social inclusion (Lecoutere et al., 2019). Understanding why DETs have (or have not) been used may help practitioners develop DETs for increased uptake and positive impacts in LMICs.

Publicly available literature contains incomplete evidence on what it takes to facilitate large-scale and equitable DET uptake in LMICs. A systematic scoping review found 243 studies (peer-reviewed and grey) that collectively evidenced 74 factors influencing uptake of digitally-enabled agricultural services in LMICs (Porciello et al., 2021). Yet three key limitations constrain the practical value of this evidence base:

1. **Outdated:** the evidence base has not kept pace with the ongoing evolution of DETs. For example, only 5% of reviewed studies focused on use of smartphone-based DETs (Porciello et al., 2021). Meanwhile, rural smartphone uptake in LMICs has grown rapidly (GSMA Intelligence, 2021); 29% of rural people in Cambodia and 19% of rural people in Ghana owned a smartphone as of 2017/2018 (Chen, 2021). This illustrates the questionable relevance of existing literature to contemporary DETs;
2. **Limited Geographic scope:** existing literature is biased towards a small number of geographies. More than 75% of studies analysing use of digitally-enabled agricultural services in LMICs focused on just seven countries, India, Kenya, Uganda, Nigeria, Ghana, Tanzania, and Ethiopia (Porciello et al., 2021);
3. **Uncertain actionability:** a wealth of studies identified pitfalls (‘a hidden or unsuspected danger or difficulty’) that constrained use of DETs, such as illiteracy preventing a farmer from actioning pest management advice delivered by SMS (Tambo et al., 2019). However, to our knowledge, no study systematically analysed how these pitfalls have been avoided.

In view of these limitations, we aimed to address two interlinked research questions. First, what pitfalls have commonly constrained use of DETs by farmers and extension actors in LMICs? Second, how have these pitfalls been avoided?

We addressed these research questions by collating developer and user perceptions across a diversity of geographies and DETs. Specifically, we led 92 in-depth interactions with 40 DET developers (people that have directly contributed to development of DETs in rural contexts) across 21 LMICs and 101 DET users (farmers and extension actors with direct access to a mobile phone) in Eastern Bihar (India). Semi-structured qualitative methods were used to facilitate flexible and in-depth responses across a diversity of contexts, contemporary DETs and approaches used to make them useful. Such findings (synthesised in a practical framework) may help DET funders and developers facilitate more useable, useful and positively impactful DETs.

## 2 Conceptual framework

We developed a DET user journey framework to structure the study. This framework delineates use of DETs into three critical steps:

- **Step 1) Access interface:** defined as accessing the digital platform that supports the DET. For example, finding and opening a video about a novel fall armyworm management practice.
- **Step 2) Access content:** defined as accessing or exchanging information or knowledge within the DET. For example, understanding the fall armyworm video (or commenting on it within YouTube).
- **Step 3) Change behaviour:** defined as acting differently as a result of using the DET. For example, discussing the fall armyworm management practice from the video with a neighbour or experimenting with the practice on-farm.

We considered applying well-recognised conceptual frameworks like the Technology Acceptance Model (Davis, 1989), Unified theory of acceptance and use of technologies (Venkatesh et al., 2003), Diffusion of Innovations (Rogers et al., 2014) or frameworks applying Sen's capability approach (Roberts et al., 2019). Some of these theoretical frameworks have proven explanatory power for analysing factors influencing uptake of DETs (Voutier et al., 2020; Rose et al., 2016; Alemu and Negash, 2015). However, we were aiming to synthesise perceptions of developers and users and concluded a less abstract framework would align more comfortably with these worldviews. The tangibility of our DET user journey framework was also attractive for making research findings accessible for a broad range of research users.

## 3 Methods

**Fig. 1:**
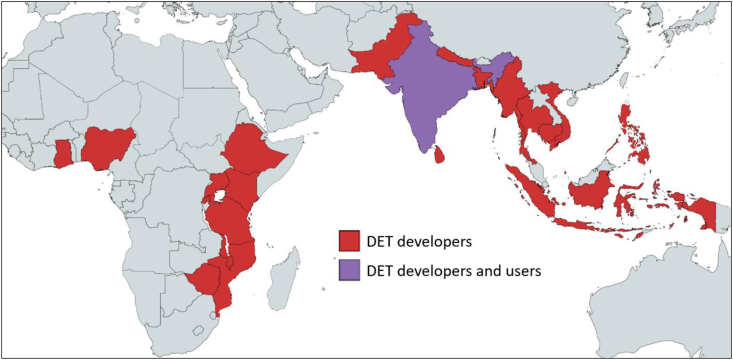
Countries represented by interviewed digital extension tool (DET) developers and users. Collectively, interviewed DET developers had worked as DET developers across ten countries in Sub-Saharan Africa (Ethiopia, Ghana, Kenya, Malawi, Mozambique, Nigeria, Rwanda, Tanzania, Uganda and Zimbabwe), five countries in South Asia (Bangladesh, India, Nepal, Pakistan and Sri Lanka) and six countries in Southeast Asia (Cambodia, Indonesia, Myanmar, Philippines, Thailand and Vietnam). Collectively, interviewed DET users had worked as DET users across four villages in Eastern Bihar (India).

We facilitated and analysed 92 semi-structured interviews (SSIs) and focus group discussions (FGDs) with 40 DET developers and 101 DET users (Table 1). Collectively, interviewed DETs developers had worked across 21 LMICs and interviewed DET users were based across four villages in Eastern Bihar, India (Fig. 1). The methods used to collect and analyse qualitative data from these interactions are detailed in sections 3.1, 3.2.

**Table 1:** Overview of semi-structured interviews and focus group discussions. All semi-structured interviews were facilitated with one respondent and all focus group discussions were facilitated with 5–8 respondents. ‘DET developers’ were defined as people that have directly contributed to development of digital extension tools (DETs) in rural contexts. ‘DET users’ were defined as farmers and extension actors with direct access to a mobile phone through someone in their household (basic phone, feature phone and/or smartphone).

Respondents	Number of semi-structured interviews	Number of focus group discussions	Number of respondents
DET developers	40	0	40
DET users	42	10	101
Total	82	10	141

### 3.1 DET developer interviews

We interviewed DET developers that were fluent English speakers and had worked as DET developers in LMICs for at least six months on a full-time basis. Suitable respondents were identified using a snowball approach; initial respondents were found through personal networks and respondents were then invited to recommend other DET developers to interview.

We actively pursued diversity between interviewed DET developers, regarding both the individuals interviewed (nationality, organisation, position, age, gender) and the DETs they had contributed to (geography, value chains, digital interfaces) (Fig. 1). Ultimately 60% of interviewed DET developers identified as male and 40% identified as female.

All DET developer interviews were led by a single facilitator, in English language, using a consistent protocol over ‘Skype’ voice calls, between March and July 2019. The interview protocol was designed to introduce respondents to the study (taking care not to bias their responses), request consent for them to anonymously participate and (with informed consent) elicit their perceptions in relation to the research questions. This protocol was refined through consultation with qualitative research experts and three pilot interviews. DET developer interviews typically lasted 30–60 minutes.

DET developer interview transcripts were analysed by the interviewer through the software ‘Quirkos’ using thematic induction. Initial interpretations were (with informed consent) informally appraised for accuracy with interviewed DET developers via email (taking care to ensure respondents remained anonymous). Interpretations were then refined based on feedback from these informal appraisals.

### 3.2 DET user interviews and focus group discussions

We interviewed farmers and other extension actors that had direct access to a mobile phone through someone in their household (basic phone, feature phone and/or smartphone) and were fluent Hindi-speakers. Similar to the DET developer interviews, suitable respondents were selected using a snowball approach; initial respondents were identified through personal networks and respondents were then invited to recommend other DET users to engage with.

As for DET developers, we actively pursued a diversity of DET user respondents. We sought a diversity of ages, genders, education levels, castes, religious beliefs, roles in agricultural extension and wealth levels (Table 2). FGDs with 5–8 participants were organised around specific groups of DET users (distinguished by gender, age, caste, years of formal education, religious beliefs, role in extension and/or farm size). SSIs with individual DET users were facilitated if it was impractical to recruit sufficient respondents of a given DET user group for an FGD.

**Table 2:** Self-reported demographic characteristics of the 101 digital extension tool (DET) users from Eastern Bihar (India) that participated in the study. DET users were defined as farmers or other extension actors with access to a mobile phone. For a small minority of respondents, it was inappropriate to capture data for every demographic variable (these missing data points are not included in this table).

Demographic variable	Respondent characteristics
Age median (and range)	35 (18–70) years
Gender	30.4% female, 69.6% male
Education level (listed in order of representation)	Secondary, tertiary, primary, no formal education
Caste (listed in order of representation)	Other Backward Class (OBC), General Category (GC), Scheduled Caste (SC)
Religious belief (listed in order of representation)	Hindu, Muslim
Role in agricultural extension (listed in order of representation)	Farmer, spouse of a farmer (but not directly involved in farming), input retailer, child of a farmer, agricultural produce buyer, Government extension worker, labourer, tractor driver
Median farm size (and range) if involved in a farm	1.2 (0.068–16) hectares

All SSIs and FGDs were led in-person by two facilitators in Hindi using consistent protocols. The SSI and FGD protocols were designed to introduce respondents to the study (taking care not to bias their responses), request consent for them to anonymously participate and (with informed consent) elicit their perceptions in relation to the research questions. Draft protocols were refined through consultation with co-authors, two pilot interviews and two pilot FGDs. DET user SSIs typically lasted 30–40 minutes and FGDs typically lasted 40–60 minutes. Digital audio recordings were later translated into English and transcribed. All DET users SSIs and FGDs occurred in December 2019.

One of the interviewers and another researcher analysed the interview transcripts through the software ‘Taguette’ using thematic induction. Themes interpreted from the DET developer SSIs were analysed against themes interpreted from the DET user SSIs and FGDs. The results were then synthesised using the DET user journey framework explained in section 2.

## 4 Results

Three results emerged. First, DET developers and users collectively identified fifteen pitfalls that commonly constrained use of DETs (section 4.1). Second, DET users often made – not just found - DETs useful (developers explained 27 tactics for avoiding the identified pitfalls and users explained 20 of their own tactics) (section 4.2). Third, both developers and users advocated strongly for involving desired users in DET provision (section 4.3).

### 4.1 Fifteen pitfalls commonly constrained use of DETs (result #1)

**Fig. 2:**
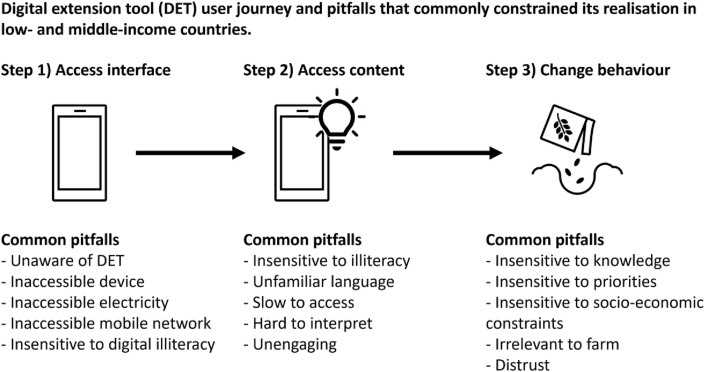
Digital extension tool (DET) developers and users identified fifteen pitfalls that commonly constrained use of DETs in LMICs. The fifteen pitfalls are organised using the ‘DET user journey’ conceptual framework explained in section 2. Five pitfalls commonly constrained DET ‘interface access’ (accessing the digital platform that supports the DET), five pitfalls commonly constrained DET ‘content access’ (accessing or exchanging information or knowledge within the DET) and five pitfalls commonly constrained ‘behaviour change’ (acting differently as a result of using the DET). Each pitfall is explained in the text below this figure.

Interviewed DET developers and users collectively identified fifteen pitfalls that commonly constrained use of DETs. Importantly, not every pitfall was identified by every interviewed DET developer and user. The fifteen pitfalls are summarised in Fig. 2 and explained below it using the DET user journey framework described in section 2.

**DET interface access** was commonly constrained by five pitfalls:

1. **Unaware of DET:** many potential users never learned of a DET's existence. “Sometimes we go to farmers and they have not even heard of [our DET]” (developer, India).
2. **Inaccessible device:** potential users were commonly unable to access necessary devices, even if they were owned by someone in the household. A 45-year-old female user lamented that her husband “takes the mobile phone with them and when they come back at night, they drink and eat and sleep. If I ask [for the phone], then they don't pay heed”. Another key nuance was devices often lacked necessary quality, including in relation to battery life, storage, processing speed, screen durability, camera functionality and counterfeit operating software.
3. **Inaccessible electricity:** electricity may have been available but not without travel and monetary costs (particularly if unavailable within the user's home). “[Farmers said] it costs me to charge my phone” (developer, Malawi).
4. **Inaccessible mobile network:** mobile networks were often available but not always fast, reliable and affordable. “Long [DET] videos will also work but then it would cost me more” (user, India, female, 32 years).
5. **Insensitive to digital illiteracy:** desired users commonly lacked experience making phone calls, navigating digital menus, saving contacts, clearing data storage, typing, downloading apps, scrolling and opening hyperlinks.

**DET content access** was commonly constrained by five pitfalls:

6. **Insensitive to illiteracy:** limited ability to read and type constrained access to DET content (not to mention DET marketing material). “At first it was all written information but we found most of the farmers were illiterate and asked for a voice over function” (developer, Cambodia)
7. **Unfamiliar language:** developers and users generally reported a strong preference for DETs to be in local languages (presuming the device supports local language fonts). Unfamiliar terms and metrics also constrained DET content access. “Farmers use the plastic cup [to measure volume]. For them, ‘5 L’ is meaningless” (developer, Pakistan).
8. **Slow to access:** numerous interviewed developers and users emphasised the importance of desired content being quick to access. “I prefer short two or 3 minutes videos. They can come straight to the point and if they are speaking about pesticide then they should quickly talk about it and finish it” (user, India, male, 38 years).
9. **Hard to interpret:** developers and users often commented on the frustration of not understanding and fear of misunderstanding DET content. “When something is in front of us, it is easier to understand things. At times on a call, it can get problematic” (user, India, female).
10. **Unengaging:** static information was unlikely to be read by desired users (let alone change their behaviour). “The video should be interesting. Not just 5 minutes but I can even watch it for half an hour if it is interesting” (user, India, male).

**DET-facilitated behaviour change** was commonly constrained by five pitfalls:

11. **Insensitive to knowledge:** DETs commonly failed to recognise experienced users' preexisting knowledge and expertise. “We know this as we are not some part-time agriculturalist but have been doing farming for the last 10 years” (user, India, male, 32 years). Similarly, DETs often failed to recognise users' ability to learn. “People misunderstand the duration of relevance of messages. Farmers go back to the platform four times and it doesn't change. Will they go back?” (developer, Kenya).
12. **Insensitive to priorities:** the value proposition of the DET (and its content) did not always align with users' priorities in regard to the decision they are facing and what they want to achieve (e.g. increase yield, reduce risk, save time). This applied to extension actors, not just farmers. “Some extension workers treat [our DET] as an additional task for them. They ask ‘what is in it for me?'” (developer, Philippines).
13. **Insensitive to socio-economic constraints:** applicability of DET content was often constrained by limited access to capital, labour, machinery, markets and recommended inputs. “Like in WhatsApp or in a message it says that this new machine has come out and you can benefit by using this machine. But in our area, I can use that only if it is available.” (user, India, male, 51 years). Similarly, applicability of DET content was also constrained by cultural constraints. “In Ghana no matter how much you push pruning, women should not be seen to be pruning” (developer, Philippines).
14. **Irrelevant to farm:** content was commonly perceived to be inapplicable to farmers' unique farming systems (including soil variation, climate variation, crop calendars and, more simply, farmers' growing the crop a DET is focused on). “If we watch videos of other places, then its climate won't match ours. We should get information according to the climate we have” (user, India, male, 48 years).
15. **Distrust:** developers and users commonly reported difficulty for users to trust information conveyed by DETs. “I saw in Rwanda there is a trust issue. [The DET] was seen as the government so the farmer didn't trust it” (developer, Rwanda).

### 4.2 DET users (not just DET developers) took responsibility for avoiding identified pitfalls (result #2)

We intentionally found how developers made DETs useful and unintentionally found that (and how) users made DETs useful. Interviewed developers implemented a variety of tactics to avoid the fifteen identified pitfalls that commonly constrained use of DETs (Appendix A). In addition, interviewed users reported mitigating the same fifteen pitfalls using notably different tactics (Appendix A). The reported user tactics are summarised below:

- **Seek community support:** Family members and peers helped users overcome device access, literacy, digital literacy and language barriers.
- **Discuss DETs with peers:** Discussing DETs and their content with peers (digitally and face-to-face) helped users become aware of DETs, trust DETs and adapt content to users' knowledge, priorities, constraints and farms.
- **Integrate information sources:** Cross-checking, integrating and choosing between multiple information sources (including videos, apps and input retailers) enabled users to interpret and appropriate content.
- **Experiment on-farm:** Testing new practices on-farm enabled farmers to adapt DETs and assess their trustworthiness.
- **Supply their own DETs:** appropriating phone calls and chat groups as DETs enabled users to time-efficiently share their farming knowledge, ask questions, entertain themselves and discuss the usefulness of other DETs.

Reported engagement in proactively making DETs useful varied across users and types of DETs. People with less education, people of lower castes and women less commonly reported making DETs useful (although women were conventionally less involved in agriculture in the studied geography). In addition, some DETs reportedly did not create room for users to make DETs useful. A female user lamented that “in a call center they speak their mind but we can't say anything from outside. Whatever they want to speak, they say and leave but I can't say anything back”. However, variability in users' making DETs useful was not analysed systematically and these weak trends should be interpreted with caution.

### 4.3 Both DET users and DET developers advocated strongly for involving users in DET provision (result #3)

Respondents advocated ubiquitously for proactively involving users in DET provision to increase DET use. This was the most common and emotionally expressed comment made by interviewed DET developers (Table 3). An interviewed developer in Kenya frustratedly observed “all these conferences, round tables blah blah blah. Experts assume farmers’ needs, design solutions for them and the solutions fail”. Similarly, multiple interviewed users stressed the importance of collaboration between developers and users. In the words of one user, “if you are making videos, then try to make these videos with the farmers where they are doing farming or show the technique that they are using. Such practical things will be more useful” (user, India, male, 29 years).

**Table 3:** Almost all interviewed digital extension tool (DET) developers independently and unpromptedly advocated for involving users in DET provision. The table synthesises comments from nine interviewed DET developers across nine LMICs (similar comments were made by most interviewed developers but not all were added to this table due to space limitations).

Sub-Saharan Africa	South Asia	Southeast Asia
“Not involving would-be users in the design remains the biggest problem for the uptake of the technology. You can't expect something magical to happen.” (developer, Kenya) “You have to include the farmer's thoughts to give them ownership and make it workable for them.” (developer, Tanzania) “A desktop design from so-called experts that may feel they know it all. Once they design it, it is not context-specific, it won't be accepted and it won't be effective.” (developer, Ethiopia)	“Completely based on farmer feedback - what they want and how they want it.” (India) “Pushing technologies that experts think should work miss technologies and practices that farmers are already doing.” (developer, Bangladesh) “You must repeatedly test what you are designing … when you do the synthesis [of user feedback], bring two farmers into the office as well.” (developer, Pakistan)	“If your goal is to reach people that aren't being reached you should go talk to these people.” (developer, Myanmar) “Too many assumptions are made [developing DETs] … when it goes to the field [farmers] don't really need it.” (developer, Indonesia) “You need a committee for two-way communication. What they need and how they can make it understandable … for us we don't see the issue [with the DET] but for them it is.” (developer, Philippines)

## 5 Discussion

We discuss each of the three core results against related literature and potential implications (sections 1, 2, 3, 4, 5). We then synthesise these findings in a one-page framework to help practitioners develop more useable and useful DETs (section 5.4). Finally, we suggest future research directions (section 5.5) and summarise conclusions (section 6).

### 5.1 Recurring pitfalls constraining use of DETs (result #1)

The fifteen pitfalls identified in this study reflect those evidenced in many other contexts. The snowball sampling and exclusion of non-English speaking DET developers may have biased our convergence on the fifteen identified pitfalls (Heckathorn, 2011). However, each of the fifteen pitfalls have been identified in at least 20 other studies (including grey literature) that evidenced factors influencing use of digitally-enabled agricultural services in LMICs (Porciello et al., 2021). Additional pitfalls have featured in other analyses, but these other pitfalls generally overlap closely with the fifteen identified in this study. For example, the ‘insensitive to socio-economic constraints’ pitfall connects directly with social network access (59 studies), capital access sensitivity (39 studies), input access sensitivity (24 studies), cultural sensitivity (15 studies), market access sensitivity (14 studies) and labour access sensitivity (3 studies) (Porciello et al., 2021). In view of this alignment with preexisting evidence and the study's broad scope (of geographies and DET types), the fifteen identified pitfalls offer a reasonably strong and transferable understanding of what constrains DET use in LMICs.

The fifteen identified pitfalls help explain why user-driven DETs are often used more than externally-driven DETs. Farmers and extension actors across Africa and Asia have commonly preferred user-driven DETs (e.g. phone calls, chat apps) over externally-driven DETs (e.g. voice message advisory services, SMS advisory services and specialised agri-apps) (Krell et al., 2021; Thar et al., 2021a; Voutier et al., 2020; Munthali et al., 2018; Butt, 2015; Rasmussen et al., 2015). This becomes more understandable in view of the fifteen identified pitfalls (Table 4). A potential implication for DET developers is ensuring externally-driven DETs are interoperable with user-driven DETs (Thar et al., 2021b).

**Table 4:**
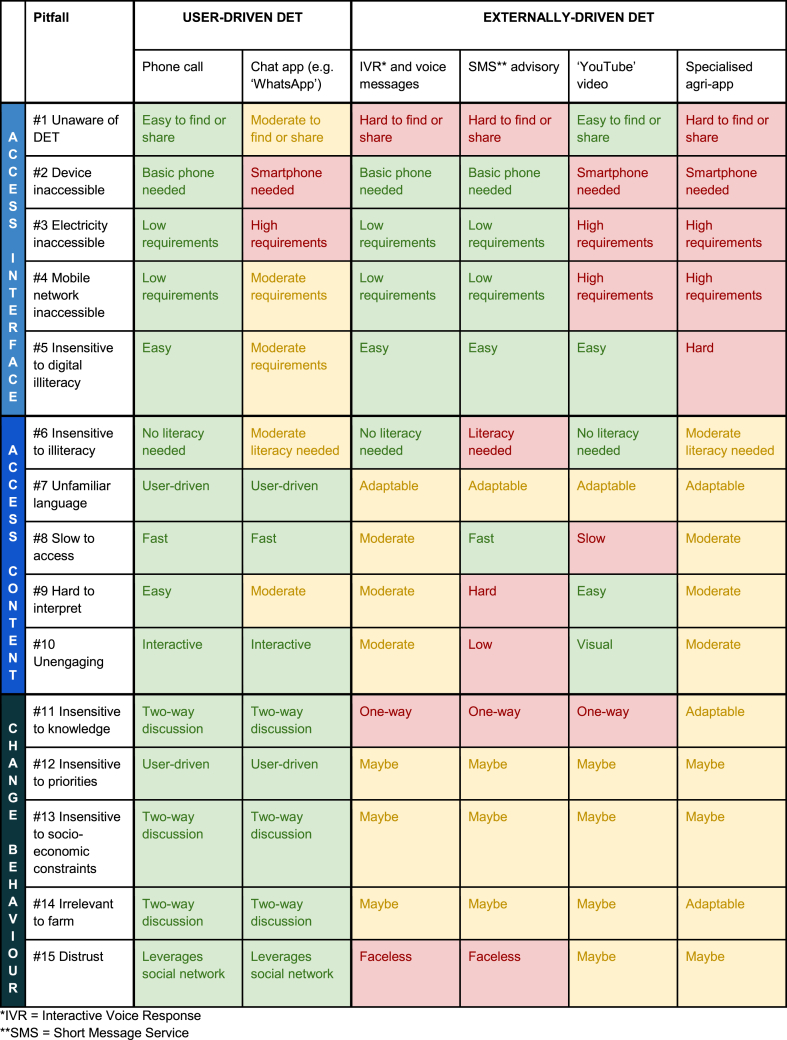
Assessment of common digital extension tool (DET) interfaces against the fifteen pitfalls that were found to commonly constrain use of DETs (building on a similar analysis by Porciello et al., 2021). Strengths are highlighted in green, weaknesses are highlighted in red and uncertainties or neutral interface attributes are highlighted in yellow. Despite coarse generalisations, the table offers partial clarity on why user-driven DET interfaces (e.g. phone calls and chat apps) are commonly used more than externally-driven DET interfaces (e.g. specialised smartphone apps).

The fifteen identified pitfalls also help explain why some user groups commonly use DETs more than others. Notwithstanding exceptions, many studies observed lower DET use by women, less educated people and less wealthy people in LMICs (Porciello et al., 2021). The fifteen identified pitfalls partially explain why these three phenomena have been observed, particularly in view of intersectionality (interaction of multiple social differences) (Table 5). Analysing the fifteen identified pitfalls against social differences may help practitioners anticipate and address barriers constraining DET use by women, less educated, less wealthy and perhaps other user groups (including ethnic groups, religious groups, older people and geographically isolated people).

**Table 5:** Application of a social exclusion lens to the fifteen pitfalls identified to commonly constrain use of digital extension tools (DETs) (building on a similar analysis by Porciello et al., 2021). References were added where specified barriers constrained specified user groups from using DETs in LMICs. References were not added where specified barriers have plausibly (without known primary evidence) constrained specified user groups from using DETs in LMICs. Despite coarse generalisations, the table offers partial clarity on why women, less educated and less wealthy people have commonly used DETs less - particularly considering interaction of these social factors.

	Pitfall	Women	Less wealthy	Less educated
**ACCESS INTERFACE**	#1 Unaware of DET	Often less information-rich social networks	Often less information-rich social networks	Often less DET awareness^1^, perhaps due to less access to DET marketing
	#2 Device inaccessible	Often less device ownership^2,3^ so higher dependence on unreliable device sharing^4,5^ or low-quality devices^6,7^	Often less cash to purchase and maintain devices of sufficient quality^8^	–
	#3 Electricity inaccessible	Often less mobility and cash to access charging stations	Often less cash to access charging stations	–
	#4 Mobile network inaccessible	Often less mobility and cash to purchase mobile network credit^8^	Often less cash to purchase mobile network credit^9,10^	–
	#5 Insensitive to digital illiteracy	Often less digital literacy^8^, perhaps due to lower device access	Often less experience with digital tools due to less ability to afford them	–
**ACCESS CONTENT**	#6 Insensitive to illiteracy	Often less literate^11^	Often less access to literacy training	Often less literate^12^
	#7 Unfamiliar language	Often less familiar with non-local languages^8^	–	Often less familiar with non-local languages and metrics
	#8 Slow to access	Often less time available due to gendered time allocations^7,13,14^	–	–
	#9 Hard to interpret	–	–	Often less familiar with abstract information^15^
	#10 Unengaging	Often less engaged in DETs that lack female role models^16,17,18^ and female intermediaries^19^	Fear of judgement may deter poorer users^4,20^	–
**CHANGE BEHAVIOUR**	#11 Insensitive to knowledge	–	–	–
	#12 Insensitive to priorities	Often less interested in DETs focused on ‘male’ practices like purchasing inputs^20,21^ instead of ‘female’ practices like managing home gardens^22^ and household nutrition^4,23,24,25^	Often less interested in practices that increase economic risk^4^	–
	#13 Insensitive to socio-economic constraints	Often more stringent cultural constraints^8^ and less control over household resources^17,20^	Often less access to inputs and capital^20^	–
	#14 Irrelevant to farm	–	–	–
	#15 Distrust	–	–	–

^1^Okello et al. (2014) - Kenya; ^2^Djohy et al. (2017) - Benin; ^3^Hudson et al. (2016) - India; ^4^Barnett et al. (2020) - Ghana; ^5^Schmidt et al. (2010) - Ghana; ^6^Wyche et al. (2019) - Kenya; ^7^Wyche and Olson (2018) - Kenya; ^8^Jensen (2007) - India; ^9^Lahiri et al. (2017) - India; ^10^Wyche et al. (2016) - Kenya; ^11^Gilissen et al. (2015) - Kenya/Zambia; ^12^Krone and Dannenberg (2016) - Kenya/Tanzania; ^13^AECF, 2015 - Kenya; ^14^Mwombe et al., 2014 - Kenya; ^15^Gowda and Dixit (2015) - India; ^16^Zossou et al. (2010) - Benin; ^17^Lecoutere et al. (2019) - Uganda; ^18^Cai et al. (2019) - Malawi; ^19^Zossou et al. (2021) - Nigeria; ^20^American Institute for Research (2018) - Kenya; ^21^Okello et al. (2012) - Kenya; ^22^Palmer and Darabian (2017a) - Sri Lanka; ^23^Palmer and Darabian (2017b) - Ghana; ^24^Palmer and Darabian (2017c) - Bangladesh; ^25^Palmer and Darabian (2017d) - Myanmar.

### 5.2 Users making (not just finding) DETs useful (result #2)

Users actively making, not just finding, DETs useful has been observed in many other contexts. Numerous studies observed users making DETs *useable* (Barnett et al., 2020; Djohy et al., 2017; Rasmussen et al., 2015). For example, pastoralists in Ethiopia placed their mobile phones on high objects to access weak mobile networks (Debsu et al., 2016). Numerous other studies observed users making DETs *useful* (Karubanga et al., 2019; Karanasios et al., 2018; Maredia et al., 2018; Gandhi et al., 2007). For example, farmers in Gujarat leveraged a voice-based agronomy forum to improve social status, entertain guests, share poetry and jokes, keep awake while irrigating and develop off-farm businesses (Patel et al., 2010). Finally, other studies have observed users *creating* DETs (Voutier et al., 2020; American Institute for Research, 2018; Munthali et al., 2018; Lewis et al., 2016; Shimamoto et al., 2015). For example, fisher people in Kerala adapted phone calls to create an informal and impactful market information service (Jensen, 2007). Evidently, use of DETs by farmers and other extension actors is often a creative and adaptive process.

The word ‘appropriation’ may describe DET use more accurately than the word ‘adoption’. Glover et al. (2019; 2017; 2016) critiques how the concept of adoption (or at least the prevalent Rogers (2003) conceptualisation) inaccurately implies that smallholder farmers passively receive and deploy agricultural innovations as fixed packages. This conceptualisation does not account for smallholder farmers actively creating and adjusting agricultural innovations to align with their existing priorities and capabilities (Appendix A; Bouwman et al., 2021; Klerkx et al., 2012b; Douthwaite et al., 2001). “In other words, technology is something people do, make or remake, not something they receive or adopt” (Glover et al., 2016). Describing DET use as ‘appropriation’ perceivably accounts for this reality more accurately than the word ‘adoption’. This reinterpretation has practical (not just semantic) importance.

Designing DETs for appropriation (not just adoption) has facilitated increased DET use. Interviewed developers and at least 40 other empirical studies found user-led discussion facilitated use of digitally-enabled agricultural services (Porciello et al., 2021). For example, an interviewed DET developer in Ethiopia sent different and complementary SMS messages to different phones in the same community to support user-led discussions; “the diversity of messages is creating spillovers, sparking discussion amongst farmers” (Appendix A). Supporting user-led discussions does not appear to be the only way to ‘create room’ for user appropriation of DETs. Other practical mechanisms include making DETs shareable via ‘Bluetooth’ (Sousa et al., 2019; Maredia et al., 2018), making DETs interoperable with chat apps (e.g. through shareable links or application programming interfaces - APIs) (Table 4; Thar et al., 2021b) and offering choices within DETs (PAD, 2019). For example, a developer in Kenya interpreted that their DET's unadaptable fertiliser recommendations were generally rejected by farmers as irrelevant to their farms and economic constraints. The developer reflected that if they started again they would “provide a few options and let the farmer choose the best of them” (Appendix A). These examples illustrate that designing DETs that ‘create room’ for active appropriation (not just passive adoption) creates meaningful opportunities to increase DET use.

### 5.3 Involving users in DET provision (result #3)

More than 25 empirical studies in LMICs found involving users in DET provision facilitated use of these DETs (Porciello et al., 2021 and references therein; Ortiz-Crespo et al., 2020). Our study contributes to this evidence base in three ways. First, we identified common pitfalls that are difficult to avoid without user involvement (e.g. we would expect a DET is more likely to be insensitive to users’ knowledge, priorities and socio-economic constraints if no users were involved in the process of providing the DET). Second, we found DET users often acted as DET developers (a practice that may be facilitated by DET developers proactively involving users in DET provision). Third, we synthesised perceptions of experienced DET developers and users that independently, unpromptedly and almost unanimously advocated for involving users in DET provision to facilitate DET use (Table 3). In view of these three findings (and the referenced literature), it appears user involvement in DET provision is central to facilitating use of DETs in LMICs.

### 5.4 Synthesis

Through unsystematic collaboration with more than forty DET funders and developers, we distilled key findings into a one-page framework to help these practitioners anticipate and address weaknesses of proposed DETs (Table 6). The framework is designed to complement (not substitute) user involvement in DET provision. The framework is focused exclusively on DET use and does not directly address scalability, commercial sustainability and downstream development impacts (positive or negative).

**Table 6:** Framework for anticipating and avoiding pitfalls that may constrain use of a digital extension tool (DET). This framework attempts to summarise and interpret the study's findings from the lens of a DET developer. The framework was developed informally by interpreting study results in discussion with interviewed DET developers.

	Potential pitfall	Supporting questions
**ACCESS INTERFACE**	#1 Unaware of DET	**Developer-led marketing:** How will the DET be marketed? **User-led marketing:** Can users easily share the DET (e.g. via Facebook)?
	#2 Device inaccessible	**Accessibility within households:** Who can/can't access required devices? **Device quality:** are accessible devices of sufficient quality to use DET (including operating software, durability, screen size, processing speed)?
	#3 Electricity inaccessible	**Digital suitability:** Can desired users access electricity with limited monetary and travel costs?
	#4 Mobile network inaccessible	**Interface suitability:** is the chosen DET interface (e.g. ‘YouTube’ video) appropriate for the mobile network reliability, speed and affordability?
	#5 Insensitive to digital illiteracy	**Interface familiarity:** Do desired users already use the chosen interface? **Interoperability:** Is the interface shareable on chat apps (e.g. ‘WhatsApp’)?
**ACCESS CONTENT**	#6 Insensitive to illiteracy	**Audio-visuals:** Is reading or typing required to use the DET? **Voice command:** Is the DET findable using voice command?
	#7 Unfamiliar language	**Language:** Can the DET offer local language? **Terms/Metrics:** Can the DET offer local terms and metrics?
	#8 Slow to access	**First use:** How long does it take for users to access benefits? **Referability:** Can desired content be easily referred to on-demand?
	#9 Hard to interpret	**Visual:** Is the content visual (or at least visualisable)? **Simplicity:** Is the content intuitive to desired users?
	#10 Unengaging	**Enjoyment:** Can DET use involve games, stories, humour, visuals or human interaction?
**CHANGE BEHAVIOUR**	#11 Insensitive to knowledge	**User knowledge:** does the DET include (or at least adapt to) users' preexisting knowledge? **Updating:** is the content updated (to account for user learning)?
	#12 Insensitive to priorities	**Who prioritises:** are the DET priorities (e.g. increased yield, reduced risk) set by users or others?
	#13 Insensitive to socio-economic constraints	**Choice:** does the DET provide users with options? **Discussion:** does the DET support discussion (within or outside the DET)?
	#14 Irrelevant to farm	**Localisation:** can the DET be adapted to local soils, climates, agronomic practices and crop calendars?
	#15 Distrust	**Branding:** is the DET branding familiar and trusted? **Testability:** is the DET content testable on a small-scale?

### 5.5 Future research directions

We suggest three directions for future research:

1. Evaluate the reliability and applicability of our framework for anticipating and avoiding pitfalls that may constrain use of DETs (Table 6). Quantitative empirical approaches include testing what identified pitfalls are most predictive of DET use metrics (such as number of installs, views, user ratings and positive reviews of DET apps on the Google Play Store and farming videos on YouTube). Qualitative empirical approaches include testing application of the framework with DET developers. Theoretical approaches include assessing our framework against other frameworks that explain use of DETs or technologies more generally.
2. Analyse conditions under which users can make (not just find) DETs useful. The extent to which users made DETs useful appeared variable across DETs and user groups. Additionally, only DET users in Eastern Bihar were included in the study. These considerations invite questions about the conditions (in relation to DET affordances, social structures and other factors) that facilitate and constrain the practice of users making DETs useful.
3. Analyse constraints to user involvement in DET provision. If ‘why involve users’ has been addressed, ‘why aren't users always involved’ may be a logical and important follow-up question, particularly in view of restrictions created by the COVID-19 pandemic (Chander and Rathod, 2020).

## 6 Conclusions

We facilitated and analysed 92 qualitative interviews and focus group discussions with DET developers and users and we arrived at three findings:

1. Fifteen pitfalls have repeatedly constrained use of DETs: five pitfalls for accessing the digital interface, five pitfalls for accessing the content and five pitfalls for influencing behaviour (Fig. 2). These fifteen pitfalls help us understand why user-driven DET interfaces (e.g. phone calls, chat apps) have commonly been used more than externally-driven DET interfaces (e.g. specialised smartphone apps) (Table 4). The fifteen pitfalls also help us understand why women, less educated and less wealthy people have commonly used DETs less (Table 5).
2. DET users often made (not just found) DETs useful for themselves and others. Specifically, users proactively made DETs useable, made DETs useful and created their own DETs. This suggests the word ‘appropriation’ conceptualises DET use more accurately and helpfully than the word ‘adoption’ (section 5.2).
3. Proactively involving users in DET provision appears central to increasing DET use. DET developers and users advocated ubiquitously, independently and unpromptedly for this practice (Table 3).

We infer DET funders and designers make more useful DETs when acknowledging desired users as fellow DET developers - either by directly collaborating with users in DET design or by designing adaptable DETs that ‘create room’ for users to appropriate DETs. We hope these conclusions (synthesised in Table 6) will help practitioners develop more useable, useful and positively impactful DETs in LIMCs.

## Declaration of competing interest

The authors declare that they have no known competing financial interests or personal relationships that could have appeared to influence the work reported in this paper.
