# Supplementary material for: How have smallholder farmers used digital extension tools? Developer and user voices from Sub-Saharan Africa, South Asia and Southeast Asia
Source: Glob Food Sec. 2022 Mar;32:100577. doi: 10.1016/j.gfs.2021.100577 (PMC8907870; doi:10.1016/j.gfs.2021.100577)
Supplement: Multimedia component 1 [file mmc1.docx]

COLOUR INTENDED ON WEB AND IN PRINT

**Appendix 1.** Developers’ and users’ reported tactics for avoiding identified pitfalls.

|  | **Pitfall** | **Developer tactics** | **User tactics** |
| --- | --- | --- | --- |
| **A**  **C**  **C**  **E**  **S**  **S**  **I**  **N**  **T**  **E**  **R**  **F**  **A**  **C**  **E** | #1 Unaware of DET | **Marketing:** “We run ads on TV channels” (developer, Sri Lanka) | **Word-of-mouth:** “Some boy must have introduced me to this app in a discussion.” (farmer, male, 68 years) |
|  | #2 Device inaccessible | **Careful interface selection:** “An app might look great to donors but only 10% of the farmers can use it.” (developer, Ethiopia) | **Peer support:** “I get [farming] information from my brother as he has a smartphone and he uses [the] internet.” (farmer, female, 32 years) |
|  | #3 Electricity inaccessible | **Avoid context:** “If you don’t have that foundation [of electricity access], you’re staring down the barrel.” (developer, Malawi) | No data |
|  | #4 Mobile network inaccessible | **Free airtime:** “we make sure we pay for all the interaction and take away the burden from the farmers.” (developer, Uganda)  **Offline access:** “Most of the things must usable offline.” (developer, Indonesia) | **Find mobile network:** “There is some network problem at home but if we go on the road or near the canal, then there are no such issues.” (farmer, male, 38 years) |
|  | #5 Insensitive to digital illiteracy | **Human support:** “You’re set up to fail if you give it to farmers without support.” (developer, Mozambique)  **User testing:** “you don’t think about these things until you sit next to farmers, watch them interact with an SMS and think ‘wow, that’s annoying’.” (developer, Uganda)  **Integrate or imitate familiar interfaces:** “We used channels farmers are already addicted to” (developer, India) | **Peer support:** “I don't have other apps on my phone. But, others have it so I watch it there.” (farmer, male, 38 years)  **Children support:** “I will tell my son to download this [pest identification smartphone app] on the phone.” (farmer, male)  **Self-education:** “I think slowly, I will learn to use my mobile phone on my own.” (farmer, male, 27 years) |
| **A**  **C**  **C**  **E**  **S**  **S**  **C**  **O**  **N**  **T**  **E**  **N**  **T** | #6 Insensitive to illiteracy | **Audio-visuals:** “We first tried SMS but farmers couldn’t make sense of it so we did [Interactive Voice Response].” (developer, Ghana) | **Voice-command:** I say it out loud (voice command) that I want such a video and it opens the video.” (farmer, female, 32 years)  **Community support:** “If I am not able to read it then I tell my son to check what it is and then he says that this is about the weather.” (farmer, female) |
|  | #7 Unfamiliar language | **Use local language(s):** “It is a shame when English speakers develop services in English.” (developer, Ghana)  **Offer choices:** “To give you an example of expensive mistakes, [a developer] built a solution in one language - English.” (developer, Kenya) | **Community support:** “If it is in Hindi, then I can read it but if it is in English then I tell others to read it.” (farmer, male)  **Leverage pictures:** “My uneducated peers focus on pictures. For example, if they see the picture of YouTube on the phone, they will know that on clicking it, they will be able to see videos.” (farmer, male, 27 years) |
|  | #8 Slow to access | **Prioritise content:** “Give them what they really really need, not just what they want. Then graduate to the next one.” (developer, Kenya)  **Avoid clunky registration processes:** “Build up the [user] profile gradually.” (developer, Malawi) | **Leverage chat groups:** “I mostly use WhatsApp as I can directly talk or if there is any problem then I can immediately get a solution.” (farmer, male) |
|  | #9 Hard to interpret | **Visual content:** “Effective communication is words, sounds, facial expressions, gestures. The video enables you to capture that.” (developer, Tanzania)  **Visualisable content:** “Wake up in the morning, go to your farm, get to your row and check for the pests under the leaf.” (developer, Kenya).  **Simplify:** “Even the extension workers were confused about the content.” (developer, Philippines) | **Clarify by cross-checking other information sources:** “Who do you like more, talking to an experienced farmer or talking with the Kisan Call Centre?  With both… we need to speak to both to understand what both of them have to say” (farmer, male) |
|  | #10 Unengaging | **Market benefits:** “If you do this you prevent this. Loss aversion will speak to them most closely.” (developer, Kenya)  **Gamification:** “Creating tests and quizzes and farmers get a free seed voucher if they pass.” (developer, Tanzania)  **Stories:** “We had four characters… the wife talks about livestock and nutrition, the son talks about tech, the dad about traditional practices. We made it like a conversation.” (developer, Pakistan)  **Support discussion:** “diversity of [complementary SMS advisory] messages is creating spillovers, sparking discussion amongst farmers.” (developer, Ethiopia) | **Make fun user-led DETs:** “We can get farming related information from [our WhatsApp chat groups] and also we use it for our own entertainment.” (farmer, male, 38) |
| **C**  **H**  **A**  **N**  **G**  **E**  **B**  **E**  **H A**  **V**  **IOU**  **R** | #11 Insensitive to knowledge | **User testing:** “You constantly need to be in touch with the farmers to keep it relevant.” (developer, Kenya)  **Focus on novel challenges:** “Pest and disease changes every year... Farmers have not had experience and need help. Sometimes farmers don't believe us about fertiliser because they have been doing that for many years.” (developer, Indonesia). | **Combine information with knowledge:** “I have heard about this [prescriptive fertiliser recommendation] app. But mainly we get weather related information from the ‘accuweather’ app. Based on the weather and the knowledge that we have about the soil, we know what to do.” (farmer, male) |
|  | #12 Insensitive to priorities | **User testing:** “We allow people to vote on the crop they want [information for].” (developer, Tanzania)  **Market benefits:** “We marketed the value proposition [of our DET] as crop security and family health.” (developer, Sri Lanka)  **Distinguish desired users:** “poorer farmers want to know about staples, business farmers want to know about profitable crops.” (developer, Malawi) | **User-led searching:** “If we want to cultivate a new crop, then we search for it on YouTube and do its cultivation. In the same way any other information that I am seeking I can find it on YouTube.” (farmer, male)  **Filter information:** “Those [SMS messages] which are important I read and those which are not important I don't read them.” (farmer, female) |
|  | #13 Insensitive to socio-economic  constraints | **User testing:** “Our tool told the farmers what their fields need but the farmers wanted to know where to invest their budget… how much do they want to invest, we need to start with that.” (developer, Kenya)  **Developer-led service bundling:** “We use the driver of the value chain [to market the DET]. For example, the exporter that takes their produce. The farmer is willing to adopt because he knows the exporter will buy the produce.” (developer, Ghana) | **User-led service bundling:** “I have saved the helpline number 1511 on my phone and have contacted them for arming related information. I also use the Kisan Suvidha app to get information about market rate or what time to sow seeds or weather forecast related stuff from it.” (farmer, male, 32 years) |
|  | #14 Irrelevant to farm | **Provide options:** “[The fertilizer] recommendations were good on average for the region but bad for individual fields... [If I could start again I would] provide a few options and let the farmer choose the best of them.” (developer, Kenya)  **Personalise to crop calendar:** “We tailor [timing] to the farmer using the planting date.” (developer, Bangladesh)  **Personalise to weather:** “we give agronomy advice based on the weather.” (developer, Ghana) | **Test on farm:** “If we get information from phone, we try that out and see if it was a success.” (farmer, female, 30 years)  **Use locally created DETs:** “YouTube is the best in today's time because it provides area specific information. Like my house is in Bihar so if anybody uploads video from Bihar or from neighbouring Uttar Pradesh, then videos of these places will give us better results as the weather is pretty much the same.” (farmer, male)  **Filter information:** “whatever information suits my area, I use them and whatever that does not suit our area, we ignore it.” (farmer, male) |
|  | #15 Distrust | **Service human support:** “Farmers still want to have personal interaction with the extension worker.” (developer, Philippines)  **Work with trusted organisation:** “We put on the face of the implementer - the people and organisation they know.” (developer, Uganda)  **Work with trusted individual:** “We used a famous sports commentator [as the voice of an audio service] and the response rate was through the roof.” (developer, Tanzania) | **Test DETs:** “We would then buy once or twice to try and only then we can trust those apps.” (farmer, male, 48 years)  **Peer recommendation:** “We ask our next door house what they have used. So the same thing I use.” (farmer, male)  **Cross-check information:** “[The text message advice] acted like a confirmation about the pesticide that we were using as they recommended the same pesticides which we use.” (farmer, female, 40 years)  **Consider providers’ intent:** “On mobile phone messages, sometimes the company to increase its profits exaggerate the benefits of its products.” (farmer, male) |
